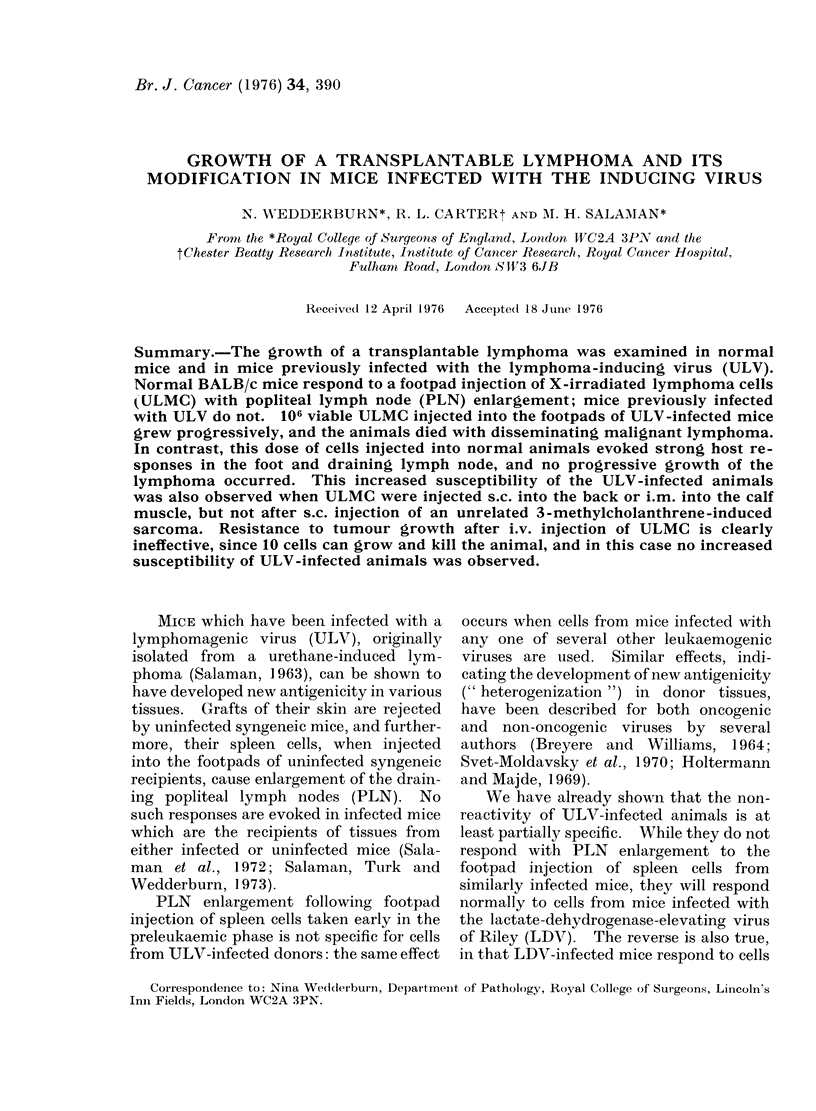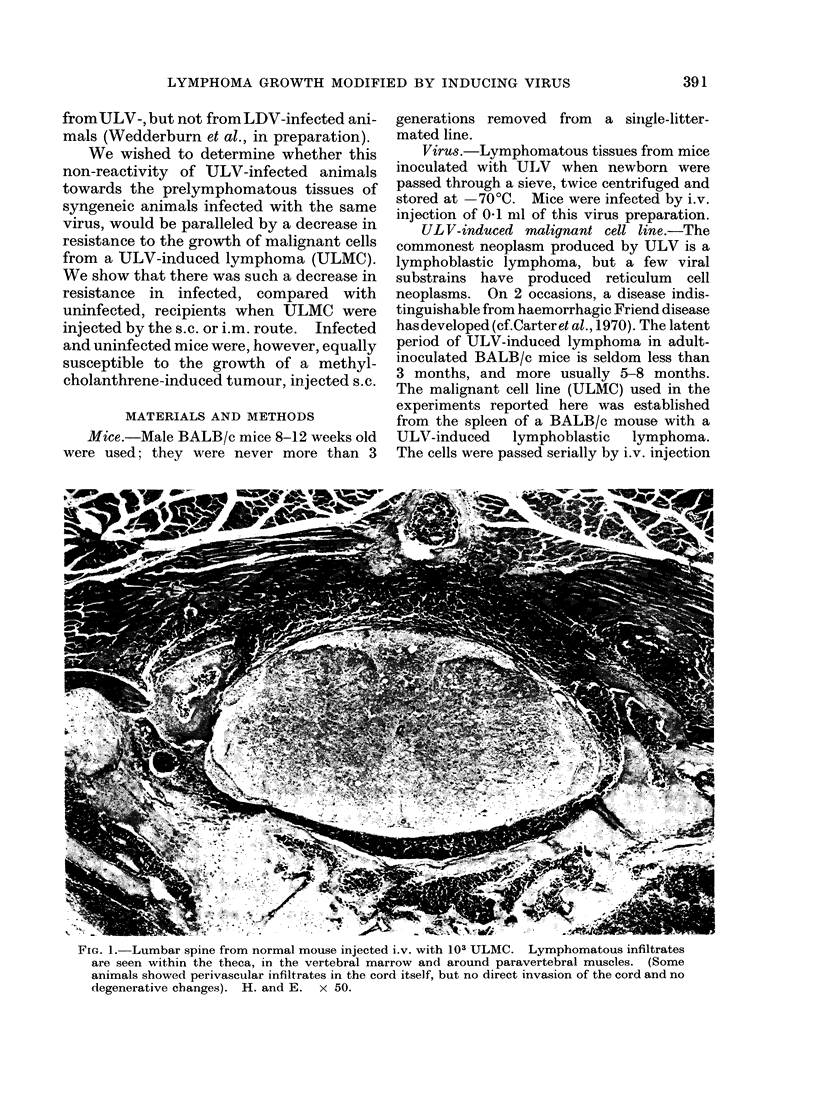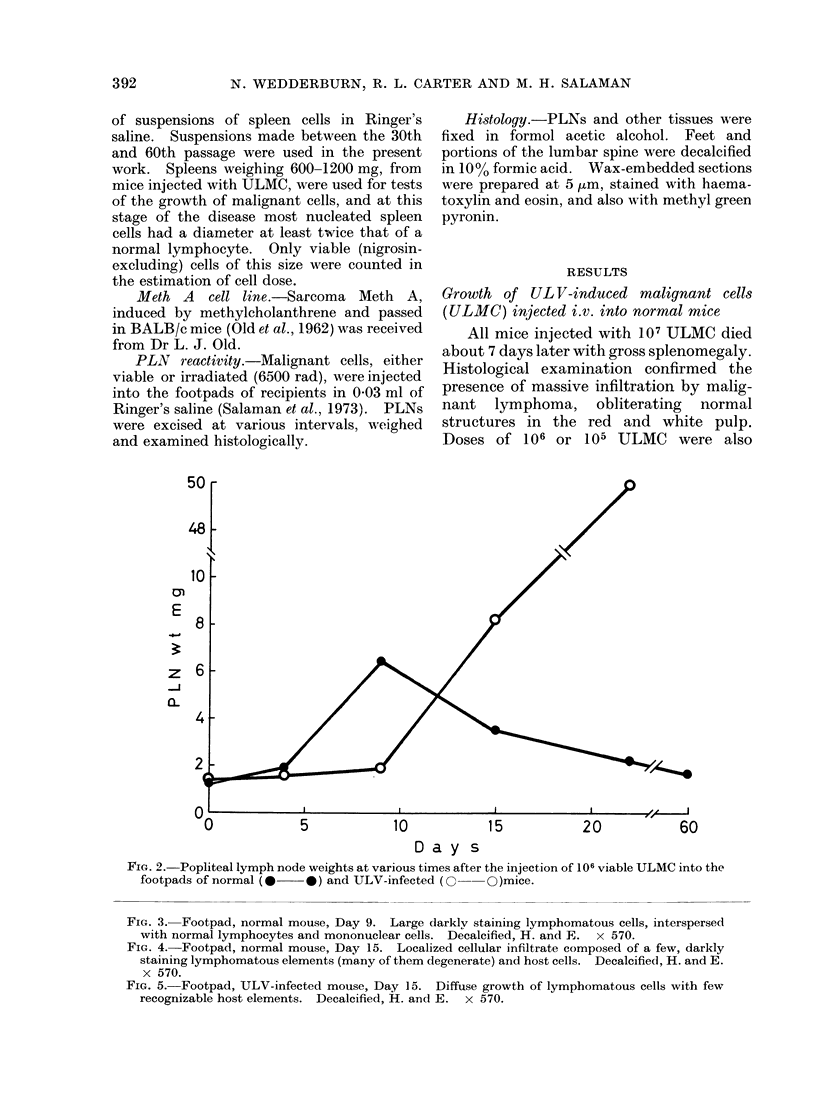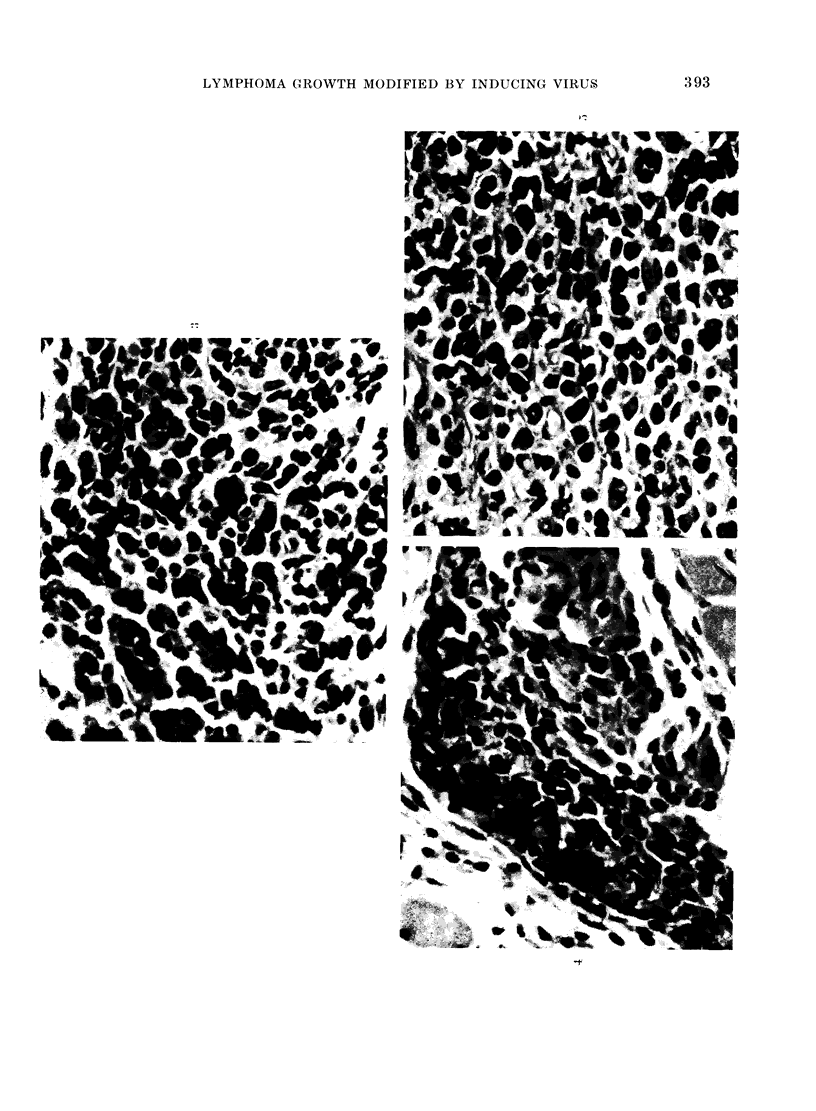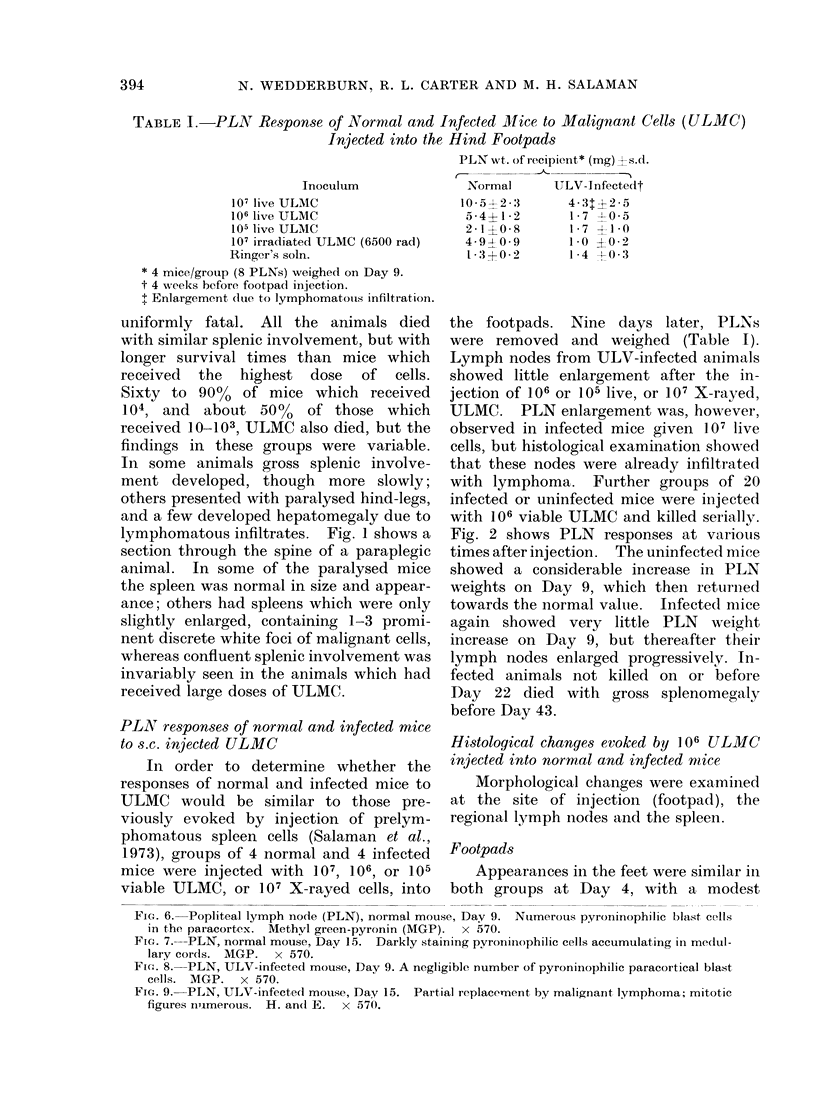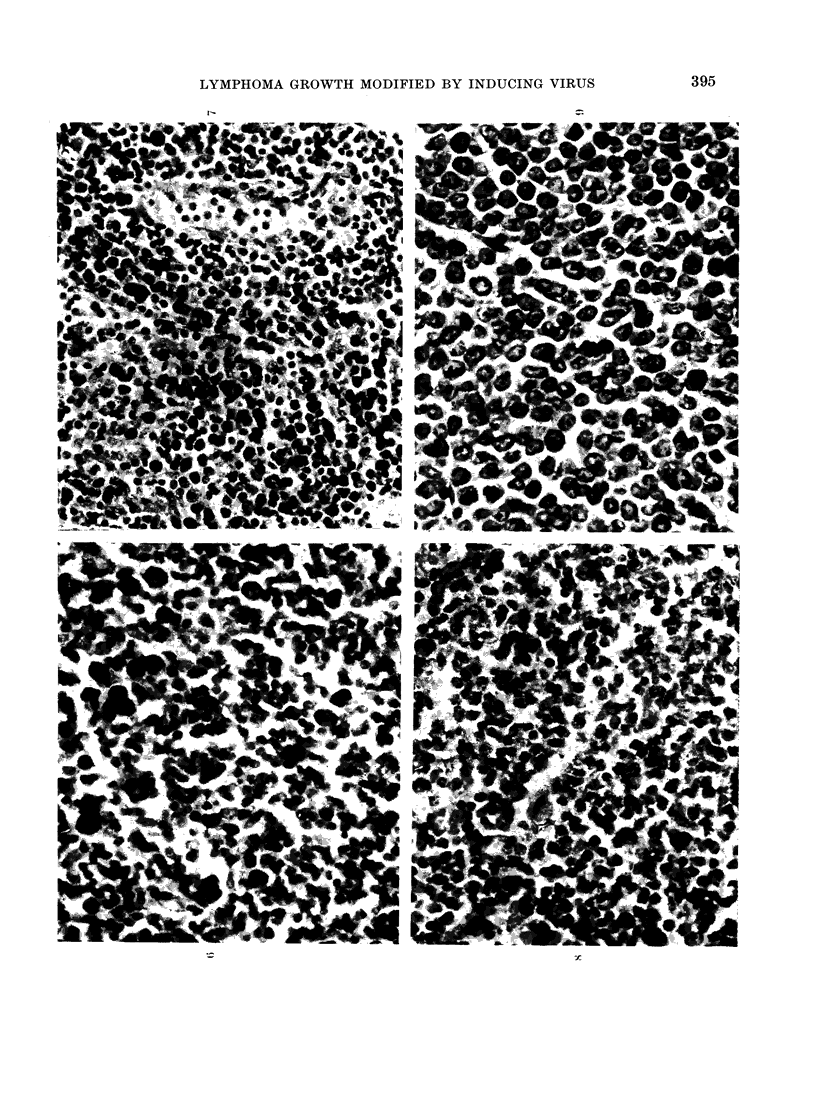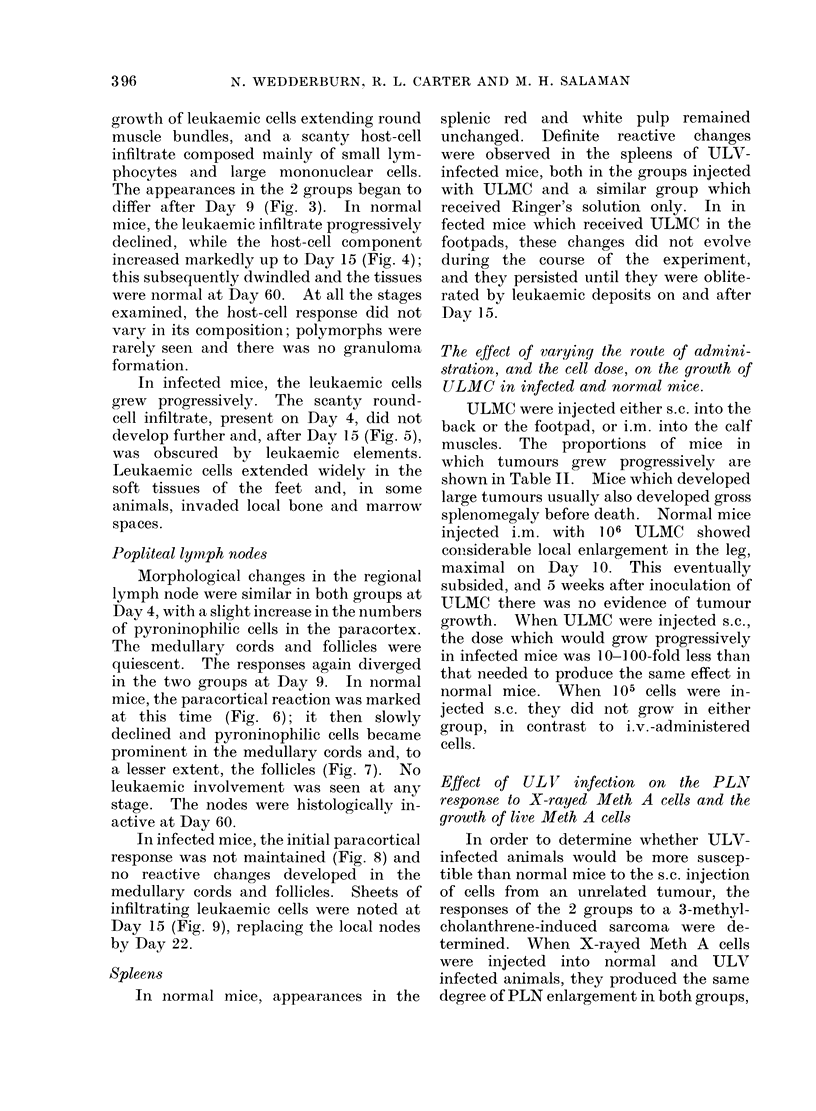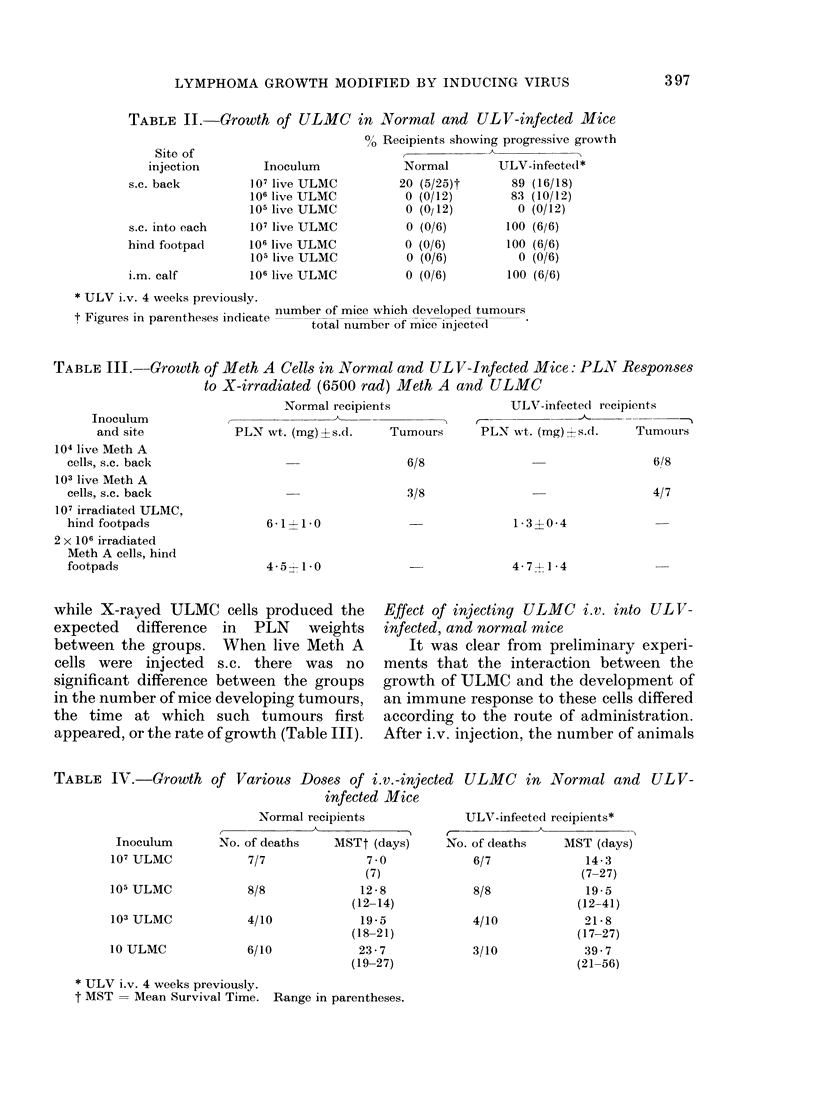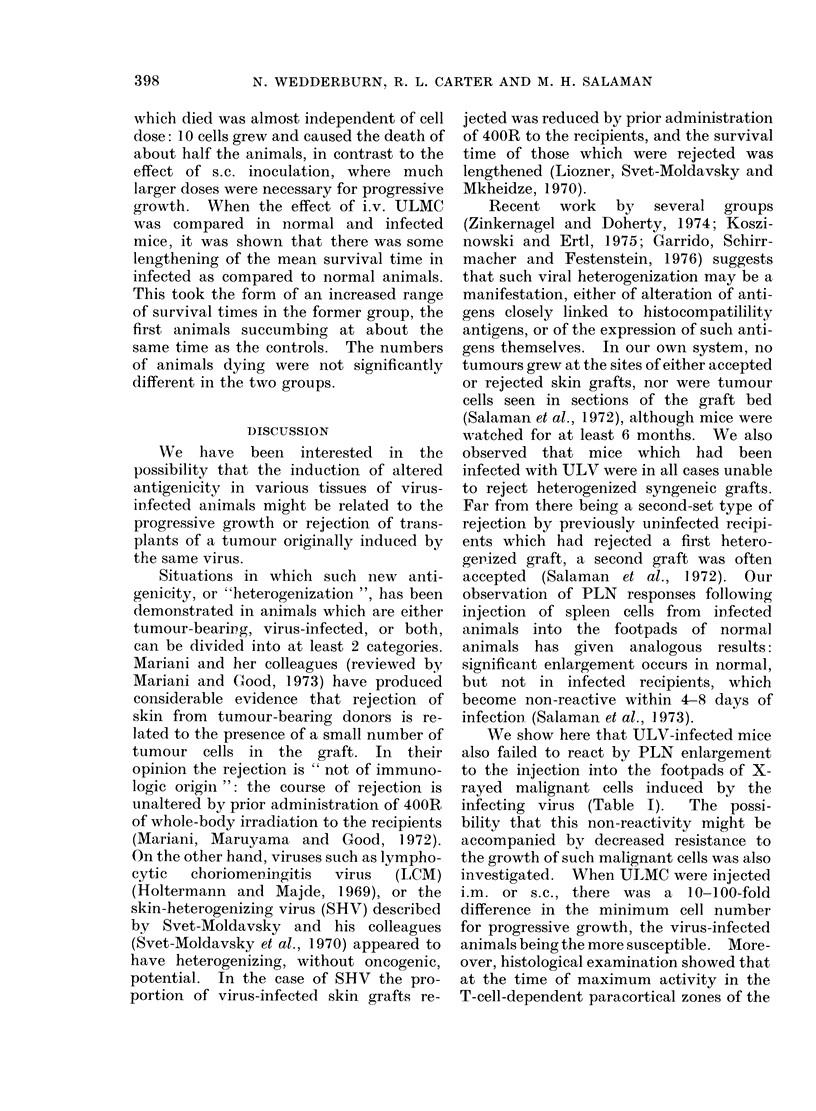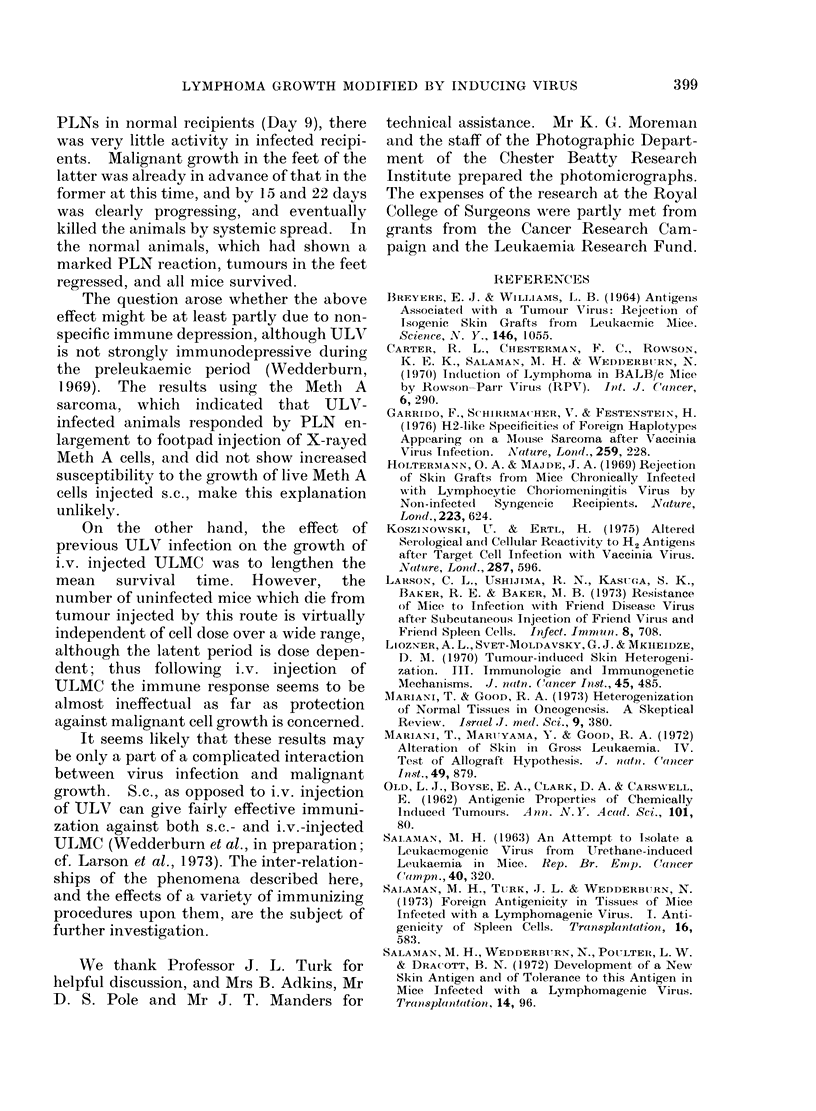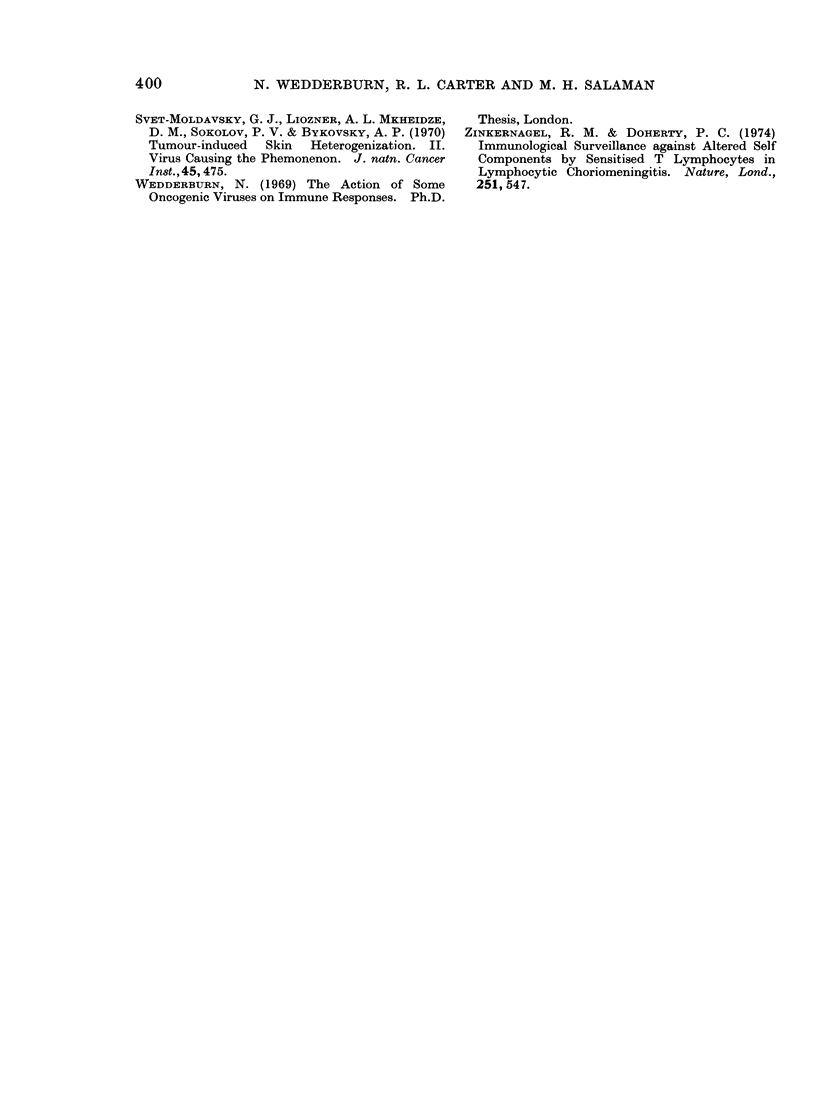# Growth of a transplantable lymphoma and its modification in mice infected with the inducing virus.

**DOI:** 10.1038/bjc.1976.182

**Published:** 1976-10

**Authors:** N. Wedderburn, R. L. Carter, M. H. Salaman

## Abstract

**Images:**


*Br. J. Cancer* (1976) **34**, 390

### GROWTH OF A TRANSPLANTABLE LYMPHOMA AND ITS MODIFICATION IN MICE INFECTED WITH THE INDUCING VIRUS

N. WEDDERBURN*, R. L. CARTER† AND M. H. SALAMAN*

*From the *Royal College of Surgeons of England, London WC2A 3PN and the †Chester Beatty Research Institute, Institute of Cancer Research, Royal Cancer Hospital, Fulham Road, London SW3 6JB*

Received 12 April 1976 Accepted 18 June 1976

**Summary.—The growth of a transplantable lymphoma was examined in normal mice and in mice previously infected with the lymphoma-inducing virus (ULV). Normal BALB/c mice respond to a footpad injection of X-irradiated lymphoma cells (ULMC) with popliteal lymph node (PLN) enlargement; mice previously infected with ULV do not. $10^6$ viable ULMC injected into the footpads of ULV-infected mice grew progressively, and the animals died with disseminating malignant lymphoma. In contrast, this dose of cells injected into normal animals evoked strong host responses in the foot and draining lymph node, and no progressive growth of the lymphoma occurred. This increased susceptibility of the ULV-infected animals was also observed when ULMC were injected s.c. into the back or i.m. into the calf muscle, but not after s.c. injection of an unrelated 3-methylcholanthrene-induced sarcoma. Resistance to tumour growth after i.v. injection of ULMC is clearly ineffective, since 10 cells can grow and kill the animal, and in this case no increased susceptibility of ULV-infected animals was observed.**

MICE which have been infected with a lymphomagenic virus (ULV), originally isolated from a urethane-induced lymphoma (Salaman, 1963), can be shown to have developed new antigenicity in various tissues. Grafts of their skin are rejected by uninfected syngeneic mice, and furthermore, their spleen cells, when injected into the footpads of uninfected syngeneic recipients, cause enlargement of the draining popliteal lymph nodes (PLN). No such responses are evoked in infected mice which are the recipients of tissues from either infected or uninfected mice (Salaman *et al.*, 1972; Salaman, Turk and Wedderburn, 1973).

PLN enlargement following footpad injection of spleen cells taken early in the preleukaemic phase is not specific for cells from ULV-infected donors: the same effect occurs when cells from mice infected with any one of several other leukaemogenic viruses are used. Similar effects, indicating the development of new antigenicity ("heterogenization") in donor tissues, have been described for both oncogenic and non-oncogenic viruses by several authors (Breyere and Williams, 1964; Svet-Moldavsky *et al.*, 1970; Holtermann and Majde, 1969).

We have already shown that the non-reactivity of ULV-infected animals is at least partially specific. While they do not respond with PLN enlargement to the footpad injection of spleen cells from similarly infected mice, they will respond normally to cells from mice infected with the lactate-dehydrogenase-elevating virus of Riley (LDV). The reverse is also true, in that LDV-infected mice respond to cells

Correspondence to: Nina Wedderburn, Department of Pathology, Royal College of Surgeons, Lincoln's Inn Fields, London WC2A 3PN.

from ULV-, but not from LDV-infected animals (Wedderburn *et al.*, in preparation).

We wished to determine whether this non-reactivity of ULV-infected animals towards the prelymphomatous tissues of syngeneic animals infected with the same virus, would be paralleled by a decrease in resistance to the growth of malignant cells from a ULV-induced lymphoma (ULMC). We show that there was such a decrease in resistance in infected, compared with uninfected, recipients when ULMC were injected by the s.c. or i.m. route. Infected and uninfected mice were, however, equally susceptible to the growth of a methylcholanthrene-induced tumour, injected s.c.

### MATERIALS AND METHODS

*Mice.*—Male BALB/c mice 8–12 weeks old were used; they were never more than 3 generations removed from a single-litter-mated line.

*Virus.*—Lymphomatous tissues from mice inoculated with ULV when newborn were passed through a sieve, twice centrifuged and stored at −70°C. Mice were infected by i.v. injection of 0·1 ml of this virus preparation.

*ULV-induced malignant cell line.*—The commonest neoplasm produced by ULV is a lymphoblastic lymphoma, but a few viral substrains have produced reticulum cell neoplasms. On 2 occasions, a disease indistinguishable from haemorrhagic Friend disease has developed (cf. Carter *et al.*, 1970). The latent period of ULV-induced lymphoma in adult-inoculated BALB/c mice is seldom less than 3 months, and more usually 5–8 months. The malignant cell line (ULMC) used in the experiments reported here was established from the spleen of a BALB/c mouse with a ULV-induced lymphoblastic lymphoma. The cells were passed serially by i.v. injection

FIG. 1.—Lumbar spine from normal mouse injected i.v. with $10^3$ ULMC. Lymphomatous infiltrates are seen within the theca, in the vertebral marrow and around paravertebral muscles. (Some animals showed perivascular infiltrates in the cord itself, but no direct invasion of the cord and no degenerative changes). H. and E. × 50.

of suspensions of spleen cells in Ringer's saline. Suspensions made between the 30th and 60th passage were used in the present work. Spleens weighing 600–1200 mg, from mice injected with ULMC, were used for tests of the growth of malignant cells, and at this stage of the disease most nucleated spleen cells had a diameter at least twice that of a normal lymphocyte. Only viable (nigrosin-excluding) cells of this size were counted in the estimation of cell dose.

*Meth A cell line.*—Sarcoma Meth A, induced by methylcholanthrene and passed in BALB/c mice (Old *et al.*, 1962) was received from Dr L. J. Old.

*PLN reactivity.*—Malignant cells, either viable or irradiated (6500 rad), were injected into the footpads of recipients in 0·03 ml of Ringer's saline (Salaman *et al.*, 1973). PLNs were excised at various intervals, weighed and examined histologically.

*Histology.*—PLNs and other tissues were fixed in formol acetic alcohol. Feet and portions of the lumbar spine were decalcified in 10% formic acid. Wax-embedded sections were prepared at 5 μm, stained with haematoxylin and eosin, and also with methyl green pyronin.

### RESULTS

### *Growth of ULV-induced malignant cells (ULMC) injected i.v. into normal mice*

All mice injected with $10^7$ ULMC died about 7 days later with gross splenomegaly. Histological examination confirmed the presence of massive infiltration by malignant lymphoma, obliterating normal structures in the red and white pulp. Doses of $10^6$ or $10^5$ ULMC were also

Fig. 2.—Popliteal lymph node weights at various times after the injection of $10^6$ viable ULMC into the footpads of normal (●——●) and ULV-infected (○——○)mice.

Fig. 3.—Footpad, normal mouse, Day 9. Large darkly staining lymphomatous cells, interspersed with normal lymphocytes and mononuclear cells. Decalcified, H. and E. × 570.

Fig. 4.—Footpad, normal mouse, Day 15. Localized cellular infiltrate composed of a few, darkly staining lymphomatous elements (many of them degenerate) and host cells. Decalcified, H. and E. × 570.

Fig. 5.—Footpad, ULV-infected mouse, Day 15. Diffuse growth of lymphomatous cells with few recognizable host elements. Decalcified, H. and E. × 570.

5

3

4

TABLE I.—*PLN Response of Normal and Infected Mice to Malignant Cells (ULMC) Injected into the Hind Footpads*

| Inoculum | PLN wt. of recipient* (mg) ± s.d. | |
|---|---|---|
| | Normal | ULV-Infected† |
| $10^7$ live ULMC | 10·5 ± 2·3 | 4·3‡ ± 2·5 |
| $10^6$ live ULMC | 5·4 ± 1·2 | 1·7 ± 0·5 |
| $10^5$ live ULMC | 2·1 ± 0·8 | 1·7 ± 1·0 |
| $10^7$ irradiated ULMC (6500 rad) | 4·9 ± 0·9 | 1·0 ± 0·2 |
| Ringer's soln. | 1·3 ± 0·2 | 1·4 ± 0·3 |

* 4 mice/group (8 PLNs) weighed on Day 9.
† 4 weeks before footpad injection.
‡ Enlargement due to lymphomatous infiltration.

uniformly fatal. All the animals died with similar splenic involvement, but with longer survival times than mice which received the highest dose of cells. Sixty to 90% of mice which received $10^4$, and about 50% of those which received $10–10^3$, ULMC also died, but the findings in these groups were variable. In some animals gross splenic involvement developed, though more slowly; others presented with paralysed hind-legs, and a few developed hepatomegaly due to lymphomatous infiltrates. Fig. 1 shows a section through the spine of a paraplegic animal. In some of the paralysed mice the spleen was normal in size and appearance; others had spleens which were only slightly enlarged, containing 1–3 prominent discrete white foci of malignant cells, whereas confluent splenic involvement was invariably seen in the animals which had received large doses of ULMC.

*PLN responses of normal and infected mice to s.c. injected ULMC*

In order to determine whether the responses of normal and infected mice to ULMC would be similar to those previously evoked by injection of prelymphomatous spleen cells (Salaman *et al.*, 1973), groups of 4 normal and 4 infected mice were injected with $10^7$, $10^6$, or $10^5$ viable ULMC, or $10^7$ X-rayed cells, into the footpads. Nine days later, PLNs were removed and weighed (Table I). Lymph nodes from ULV-infected animals showed little enlargement after the injection of $10^6$ or $10^5$ live, or $10^7$ X-rayed, ULMC. PLN enlargement was, however, observed in infected mice given $10^7$ live cells, but histological examination showed that these nodes were already infiltrated with lymphoma. Further groups of 20 infected or uninfected mice were injected with $10^6$ viable ULMC and killed serially. Fig. 2 shows PLN responses at various times after injection. The uninfected mice showed a considerable increase in PLN weights on Day 9, which then returned towards the normal value. Infected mice again showed very little PLN weight increase on Day 9, but thereafter their lymph nodes enlarged progressively. Infected animals not killed on or before Day 22 died with gross splenomegaly before Day 43.

*Histological changes evoked by $10^6$ ULMC injected into normal and infected mice*

Morphological changes were examined at the site of injection (footpad), the regional lymph nodes and the spleen.

*Footpads*

Appearances in the feet were similar in both groups at Day 4, with a modest

FIG. 6.—Popliteal lymph node (PLN), normal mouse, Day 9. Numerous pyroninophilic blast cells in the paracortex. Methyl green-pyronin (MGP). × 570.
FIG. 7.—PLN, normal mouse, Day 15. Darkly staining pyroninophilic cells accumulating in medullary cords. MGP. × 570.
FIG. 8.—PLN, ULV-infected mouse, Day 9. A negligible number of pyroninophilic paracortical blast cells. MGP. × 570.
FIG. 9.—PLN, ULV-infected mouse, Day 15. Partial replacement by malignant lymphoma; mitotic figures numerous. H. and E. × 570.

7

9

6

8

growth of leukaemic cells extending round muscle bundles, and a scanty host-cell infiltrate composed mainly of small lymphocytes and large mononuclear cells. The appearances in the 2 groups began to differ after Day 9 (Fig. 3). In normal mice, the leukaemic infiltrate progressively declined, while the host-cell component increased markedly up to Day 15 (Fig. 4); this subsequently dwindled and the tissues were normal at Day 60. At all the stages examined, the host-cell response did not vary in its composition; polymorphs were rarely seen and there was no granuloma formation.

In infected mice, the leukaemic cells grew progressively. The scanty round-cell infiltrate, present on Day 4, did not develop further and, after Day 15 (Fig. 5), was obscured by leukaemic elements. Leukaemic cells extended widely in the soft tissues of the feet and, in some animals, invaded local bone and marrow spaces.

*Popliteal lymph nodes*

Morphological changes in the regional lymph node were similar in both groups at Day 4, with a slight increase in the numbers of pyroninophilic cells in the paracortex. The medullary cords and follicles were quiescent. The responses again diverged in the two groups at Day 9. In normal mice, the paracortical reaction was marked at this time (Fig. 6); it then slowly declined and pyroninophilic cells became prominent in the medullary cords and, to a lesser extent, the follicles (Fig. 7). No leukaemic involvement was seen at any stage. The nodes were histologically inactive at Day 60.

In infected mice, the initial paracortical response was not maintained (Fig. 8) and no reactive changes developed in the medullary cords and follicles. Sheets of infiltrating leukaemic cells were noted at Day 15 (Fig. 9), replacing the local nodes by Day 22.

*Spleens*

In normal mice, appearances in the splenic red and white pulp remained unchanged. Definite reactive changes were observed in the spleens of ULV-infected mice, both in the groups injected with ULMC and a similar group which received Ringer's solution only. In in fected mice which received ULMC in the footpads, these changes did not evolve during the course of the experiment, and they persisted until they were obliterated by leukaemic deposits on and after Day 15.

*The effect of varying the route of administration, and the cell dose, on the growth of ULMC in infected and normal mice.*

ULMC were injected either s.c. into the back or the footpad, or i.m. into the calf muscles. The proportions of mice in which tumours grew progressively are shown in Table II. Mice which developed large tumours usually also developed gross splenomegaly before death. Normal mice injected i.m. with $10^6$ ULMC showed considerable local enlargement in the leg, maximal on Day 10. This eventually subsided, and 5 weeks after inoculation of ULMC there was no evidence of tumour growth. When ULMC were injected s.c., the dose which would grow progressively in infected mice was 10–100-fold less than that needed to produce the same effect in normal mice. When $10^5$ cells were injected s.c. they did not grow in either group, in contrast to i.v.-administered cells.

*Effect of ULV infection on the PLN response to X-rayed Meth A cells and the growth of live Meth A cells*

In order to determine whether ULV-infected animals would be more susceptible than normal mice to the s.c. injection of cells from an unrelated tumour, the responses of the 2 groups to a 3-methylcholanthrene-induced sarcoma were determined. When X-rayed Meth A cells were injected into normal and ULV infected animals, they produced the same degree of PLN enlargement in both groups,

TABLE II.—*Growth of ULMC in Normal and ULV-infected Mice*

| Site of injection | Inoculum | % Recipients showing progressive growth: Normal | ULV-infected* |
|---|---|---|---|
| s.c. back | $10^7$ live ULMC | 20 (5/25)† | 89 (16/18) |
| | $10^6$ live ULMC | 0 (0/12) | 83 (10/12) |
| | $10^5$ live ULMC | 0 (0/12) | 0 (0/12) |
| s.c. into each | $10^7$ live ULMC | 0 (0/6) | 100 (6/6) |
| hind footpad | $10^6$ live ULMC | 0 (0/6) | 100 (6/6) |
| | $10^5$ live ULMC | 0 (0/6) | 0 (0/6) |
| i.m. calf | $10^6$ live ULMC | 0 (0/6) | 100 (6/6) |

* ULV i.v. 4 weeks previously.

† Figures in parentheses indicate $\frac{\text{number of mice which developed tumours}}{\text{total number of mice injected}}$.

TABLE III.—*Growth of Meth A Cells in Normal and ULV-Infected Mice: PLN Responses to X-irradiated* (6500 *rad*) *Meth A and ULMC*

| Inoculum and site | Normal recipients: PLN wt. (mg) ± s.d. | Tumours | ULV-infected recipients: PLN wt. (mg) ± s.d. | Tumours |
|---|---|---|---|---|
| $10^4$ live Meth A cells, s.c. back | — | 6/8 | — | 6/8 |
| $10^3$ live Meth A cells, s.c. back | — | 3/8 | — | 4/7 |
| $10^7$ irradiated ULMC, hind footpads | 6·1 ± 1·0 | — | 1·3 ± 0·4 | — |
| $2 \times 10^6$ irradiated Meth A cells, hind footpads | 4·5 ± 1·0 | — | 4·7 ± 1·4 | — |

while X-rayed ULMC cells produced the expected difference in PLN weights between the groups. When live Meth A cells were injected s.c. there was no significant difference between the groups in the number of mice developing tumours, the time at which such tumours first appeared, or the rate of growth (Table III).

*Effect of injecting ULMC i.v. into ULV-infected, and normal mice*

It was clear from preliminary experiments that the interaction between the growth of ULMC and the development of an immune response to these cells differed according to the route of administration. After i.v. injection, the number of animals

TABLE IV.—*Growth of Various Doses of i.v.-injected ULMC in Normal and ULV-infected Mice*

| Inoculum | Normal recipients: No. of deaths | MST† (days) | ULV-infected recipients*: No. of deaths | MST (days) |
|---|---|---|---|---|
| $10^7$ ULMC | 7/7 | 7·0 (7) | 6/7 | 14·3 (7–27) |
| $10^5$ ULMC | 8/8 | 12·8 (12–14) | 8/8 | 19·5 (12–41) |
| $10^3$ ULMC | 4/10 | 19·5 (18–21) | 4/10 | 21·8 (17–27) |
| 10 ULMC | 6/10 | 23·7 (19–27) | 3/10 | 39·7 (21–56) |

* ULV i.v. 4 weeks previously.

† MST = Mean Survival Time. Range in parentheses.

which died was almost independent of cell dose: 10 cells grew and caused the death of about half the animals, in contrast to the effect of s.c. inoculation, where much larger doses were necessary for progressive growth. When the effect of i.v. ULMC was compared in normal and infected mice, it was shown that there was some lengthening of the mean survival time in infected as compared to normal animals. This took the form of an increased range of survival times in the former group, the first animals succumbing at about the same time as the controls. The numbers of animals dying were not significantly different in the two groups.

### DISCUSSION

We have been interested in the possibility that the induction of altered antigenicity in various tissues of virus-infected animals might be related to the progressive growth or rejection of transplants of a tumour originally induced by the same virus.

Situations in which such new antigenicity, or "heterogenization ", has been demonstrated in animals which are either tumour-bearing, virus-infected, or both, can be divided into at least 2 categories. Mariani and her colleagues (reviewed by Mariani and Good, 1973) have produced considerable evidence that rejection of skin from tumour-bearing donors is related to the presence of a small number of tumour cells in the graft. In their opinion the rejection is " not of immunologic origin ": the course of rejection is unaltered by prior administration of 400R of whole-body irradiation to the recipients (Mariani, Maruyama and Good, 1972). On the other hand, viruses such as lymphocytic choriomeningitis virus (LCM) (Holtermann and Majde, 1969), or the skin-heterogenizing virus (SHV) described by Svet-Moldavsky and his colleagues (Svet-Moldavsky *et al.*, 1970) appeared to have heterogenizing, without oncogenic, potential. In the case of SHV the proportion of virus-infected skin grafts rejected was reduced by prior administration of 400R to the recipients, and the survival time of those which were rejected was lengthened (Liozner, Svet-Moldavsky and Mkheidze, 1970).

Recent work by several groups (Zinkernagel and Doherty, 1974; Koszinowski and Ertl, 1975; Garrido, Schirrmacher and Festenstein, 1976) suggests that such viral heterogenization may be a manifestation, either of alteration of antigens closely linked to histocompatilility antigens, or of the expression of such antigens themselves. In our own system, no tumours grew at the sites of either accepted or rejected skin grafts, nor were tumour cells seen in sections of the graft bed (Salaman *et al.*, 1972), although mice were watched for at least 6 months. We also observed that mice which had been infected with ULV were in all cases unable to reject heterogenized syngeneic grafts. Far from there being a second-set type of rejection by previously uninfected recipients which had rejected a first heterogenized graft, a second graft was often accepted (Salaman *et al.*, 1972). Our observation of PLN responses following injection of spleen cells from infected animals into the footpads of normal animals has given analogous results: significant enlargement occurs in normal, but not in infected recipients, which become non-reactive within 4–8 days of infection (Salaman *et al.*, 1973).

We show here that ULV-infected mice also failed to react by PLN enlargement to the injection into the footpads of X-rayed malignant cells induced by the infecting virus (Table I). The possibility that this non-reactivity might be accompanied by decreased resistance to the growth of such malignant cells was also investigated. When ULMC were injected i.m. or s.c., there was a 10–100-fold difference in the minimum cell number for progressive growth, the virus-infected animals being the more susceptible. Moreover, histological examination showed that at the time of maximum activity in the T-cell-dependent paracortical zones of the

PLNs in normal recipients (Day 9), there was very little activity in infected recipients. Malignant growth in the feet of the latter was already in advance of that in the former at this time, and by 15 and 22 days was clearly progressing, and eventually killed the animals by systemic spread. In the normal animals, which had shown a marked PLN reaction, tumours in the feet regressed, and all mice survived.

The question arose whether the above effect might be at least partly due to non-specific immune depression, although ULV is not strongly immunodepressive during the preleukaemic period (Wedderburn, 1969). The results using the Meth A sarcoma, which indicated that ULV-infected animals responded by PLN enlargement to footpad injection of X-rayed Meth A cells, and did not show increased susceptibility to the growth of live Meth A cells injected s.c., make this explanation unlikely.

On the other hand, the effect of previous ULV infection on the growth of i.v. injected ULMC was to lengthen the mean survival time. However, the number of uninfected mice which die from tumour injected by this route is virtually independent of cell dose over a wide range, although the latent period is dose dependent; thus following i.v. injection of ULMC the immune response seems to be almost ineffectual as far as protection against malignant cell growth is concerned.

It seems likely that these results may be only a part of a complicated interaction between virus infection and malignant growth. S.c., as opposed to i.v. injection of ULV can give fairly effective immunization against both s.c.- and i.v.-injected ULMC (Wedderburn *et al.*, in preparation; cf. Larson *et al.*, 1973). The inter-relationships of the phenomena described here, and the effects of a variety of immunizing procedures upon them, are the subject of further investigation.

We thank Professor J. L. Turk for helpful discussion, and Mrs B. Adkins, Mr D. S. Pole and Mr J. T. Manders for technical assistance. Mr K. G. Moreman and the staff of the Photographic Department of the Chester Beatty Research Institute prepared the photomicrographs. The expenses of the research at the Royal College of Surgeons were partly met from grants from the Cancer Research Campaign and the Leukaemia Research Fund.

### REFERENCES

Breyere, E. J. & Williams, L. B. (1964) Antigens Associated with a Tumour Virus: Rejection of Isogenic Skin Grafts from Leukaemic Mice. *Science, N. Y.*, **146**, 1055.

Carter, R. L., Chesterman, F. C., Rowson, K. E. K., Salaman, M. H. & Wedderburn, N. (1970) Induction of Lymphoma in BALB/c Mice by Rowson–Parr Virus (RPV). *Int. J. Cancer*, **6**, 290.

Garrido, F., Schirrmacher, V. & Festenstein, H. (1976) H2-like Specificities of Foreign Haplotypes Appearing on a Mouse Sarcoma after Vaccinia Virus Infection. *Nature, Lond.*, **259**, 228.

Holtermann, O. A. & Majde, J. A. (1969) Rejection of Skin Grafts from Mice Chronically Infected with Lymphocytic Choriomeningitis Virus by Non-infected Syngeneic Recipients. *Nature, Lond.*, **223**, 624.

Koszinowski, U. & Ertl, H. (1975) Altered Serological and Cellular Reactivity to $H_2$ Antigens after Target Cell Infection with Vaccinia Virus. *Nature, Lond.*, **287**, 596.

Larson, C. L., Ushijima, R. N., Kasuga, S. K., Baker, R. E. & Baker, M. B. (1973) Resistance of Mice to Infection with Friend Disease Virus after Subcutaneous Injection of Friend Virus and Friend Spleen Cells. *Infect. Immun.* **8**, 708.

Liozner, A. L., Svet-Moldavsky, G. J. & Mkheidze, D. M. (1970) Tumour-induced Skin Heterogenization. III. Immunologic and Immunogenetic Mechanisms. *J. natn. Cancer Inst.*, **45**, 485.

Mariani, T. & Good, R. A. (1973) Heterogenization of Normal Tissues in Oncogenesis. A Skeptical Review. *Israel J. med. Sci.*, **9**, 380.

Mariani, T., Maruyama, Y. & Good, R. A. (1972) Alteration of Skin in Gross Leukaemia. IV. Test of Allograft Hypothesis. *J. natn. Cancer Inst.*, **49**, 879.

Old, L. J., Boyse, E. A., Clark, D. A. & Carswell, E. (1962) Antigenic Properties of Chemically Induced Tumours. *Ann. N.Y. Acad. Sci.*, **101**, 80.

Salaman, M. H. (1963) An Attempt to Isolate a Leukaemogenic Virus from Urethane-induced Leukaemia in Mice. *Rep. Br. Emp. Cancer Campn.*, **40**, 320.

Salaman, M. H., Turk, J. L. & Wedderburn, N. (1973) Foreign Antigenicity in Tissues of Mice Infected with a Lymphomagenic Virus. I. Antigenicity of Spleen Cells. *Transplantation*, **16**, 583.

Salaman, M. H., Wedderburn, N., Poulter, L. W. & Dracott, B. N. (1972) Development of a New Skin Antigen and of Tolerance to this Antigen in Mice Infected with a Lymphomagenic Virus. *Transplantation*, **14**, 96.

SVET-MOLDAVSKY, G. J., LIOZNER, A. L. MKHEIDZE, D. M., SOKOLOV, P. V. & BYKOVSKY, A. P. (1970) Tumour-induced Skin Heterogenization. II. Virus Causing the Phemonenon. *J. natn. Cancer Inst.*, **45**, 475.

WEDDERBURN, N. (1969) The Action of Some Oncogenic Viruses on Immune Responses. Ph.D. Thesis, London.

ZINKERNAGEL, R. M. & DOHERTY, P. C. (1974) Immunological Surveillance against Altered Self Components by Sensitised T Lymphocytes in Lymphocytic Choriomeningitis. *Nature, Lond.*, **251**, 547.